# Quantitative analysis of neural tissues around the optic disc after panretinal photocoagulation in patients with diabetic retinopathy

**DOI:** 10.1371/journal.pone.0186229

**Published:** 2017-10-17

**Authors:** Hyun Seung Yang, June-Gone Kim, Jae Bong Cha, Young In Yun, Jong Hoon Park, Jong Eun Woo

**Affiliations:** 1 Department of Ophthalmology, Seoul Shinsegae Eye Center, Eui Jung Bu, Gyeonggi-do, South Korea; 2 Department of Ophthalmology, University of Ulsan, College of Medicine, Asan Medical Center, Seoul, South Korea; 3 Department of Ophthalmology, University of Ulsan, College of Medicine, Ulsan University Hospital, Ulsan, South Korea; 4 Seoul National University, College of Medicine, Seoul, South Korea; Bascom Palmer Eye Institute, UNITED STATES

## Abstract

In this retrospective cross-sectional study, we quantitatively analyzed the tomographic features in the neural tissues around the optic disc in patients with diabetic retinopathy with and without panretinal photocoagulation. We analyzed 206 eyes, comprising 33 normal eyes in subjects without diabetes (group I), 30 eyes without diabetic retinopathy (group II), 66 eyes with non-proliferative diabetic retinopathy (group III), 45 eyes with panretinal photocoagulation (group IV), and 32 eyes with normal tension glaucoma (group V). Sequential images acquired using swept-source optical coherence tomography in three-dimensional mode were used to measure peripapillary retinal nerve fiber layer thickness, neuro-retinal rim thickness, anterior lamina cribrosa depth, prelaminar thickness, and thickness of the lamina cribrosa. The peripapillary retinal nerve fiber layer thickness and lamina cribrosa thickness were significantly thinner in group IV than in group III (p = 0.019 and p < 0.001). However, there was no significant difference in rim thickness, anterior lamina cribrosa depth, or prelaminar thickness between groups III and IV (p = 0.307, p = 0.877, and p = 0.212). Multivariate analysis revealed that time since panretinal photocoagulation and thickness of the lamina cribrosa had a significant effect on peripapillary retinal nerve fiber layer thickness (p < 0.001 and p = 0.014). In group IV, there was a negative correlation between time elapsed since panretinal photocoagulation and peripapillary retinal nerve fiber layer thickness, rim thickness, and thickness of the lamina cribrosa (r = -0.765, r = -0.490, and r = -0.419), but no correlation with prelaminar thickness or anterior lamina cribrosa depth (r = 0.104 and r = -0.171). Panretinal photocoagulation may be related to thinning of the peripapillary retinal nerve fiber layer, rim thickness, and lamina cribrosa, but not prelaminar thickness or anterior lamina cribrosa depth. These features are different from the peripapillary features of eyes with typical normal tension glaucoma.

## Introduction

Diabetes mellitus is a systemic disease with well-known micro-vascular and macro-vascular complications [1]. Diabetic retinopathy is one of the most common micro-vascular complications and a leading cause of blindness in both developed and developing countries [2]. Although the relationship between diabetes and development or progression of glaucoma is not completely clear, it is often difficult to clinically discriminate diabetes-related changes in the optic disc from the changes induced by panretinal photocoagulation (PRP) [1–4].

Population-based studies [5–7] have indicated that the prevalence of open-angle glaucoma in the diabetic patient population is at least twice that in the non-diabetic general population. However, the Framingham Eye Study [8] failed to replicate this finding. Studies on the progression of glaucoma have also reported inconsistent results. In particular, the Collaborative Initial Glaucoma Treatment Study (CIGTS) and Advanced Glaucoma Intervention Study (AGIS) suggested that diabetes may be a risk factor for progression of glaucoma, while the CNTGS (Collaborative Normal Tension Glaucoma Study) and EMGT (Early Manifest Glaucoma Trial) failed to show this correlation [9].

Therefore, it appears unlikely that development and/or progression of glaucoma alone accounts for the morphologic changes observed at the optic disc in patients with diabetes. These morphologic changes are often reported as non-glaucomatous optic neuropathy, which is characterized by optic disc pallor and diffuse defects in the retinal nerve fiber layer (RNFL), rather than glaucomatous changes in the optic nerve, which are characterized by a decreased neuro-retinal rim area and focal notching [3]. These changes have also been reported to be more remarkable after PRP [3,10,11]. Accordingly, previous studies have focused largely on the visual field, changes in the shape of the optic nerve head, and thinning of the RNFL. Further, the previous studies used subjective non-quantitative measurement techniques, allowing confounding factors, such as non-specific visual field defects, diabetic macular edema, and pale disc color, caused primarily by diabetic retinopathy, to affect the results. Therefore, there is a need for more objective and quantitative measurement methods. The development of spectral-domain optical coherence tomography (SD-OCT) and swept-source optical coherence tomography (SS-OCT) has enabled better and more detailed visualization of the anatomic features around the optic disc and both the anterior and posterior surfaces of the lamina cribrosa. Although it is hoped that identification of a pattern of structural changes in the optic disc and adjacent areas may help unravel the changes that occur in the optic disc in patients with diabetes, no systematic or quantitative investigation of these changes, whether along the disease severity continuum or after PRP, has been conducted using SS-OCT to date.

Therefore, the aims of this study were to identify the tomographic characteristics observed at the optic disc in patients with diabetic retinopathy treated or not by PRP and to compare the data with those obtained in patients with normal tension glaucoma (NTG).

## Materials and methods

In this cross-sectional study, we retrospectively reviewed the charts of diabetic patients diagnosed with/without diabetic retinopathy and graded using slit-lamp examination with pupil dilation by two retinal specialists (HSY, JEW) at our Diabetic Retina Clinic, Ulsan University Hospital, between June 1, 2014 and January 31, 2015. If required, fluorescence angiography was performed to assess the severity of diabetic retinopathy. According to the Early Treatment Diabetic Retinopathy Study (ETDRS) scale, the severity of diabetic retinopathy was graded as 1 (no diabetic retinopathy), 2 (non-proliferative diabetic retinopathy [NPDR]), or 3 (proliferative diabetic retinopathy). All procedures related to this study were performed in accordance with the 1975 Helsinki Declaration and its 1983 revision and were approved by the institutional review board at Ulsan University Hospital in South Korea (approval number UUH IRB 2015-04-017). The requirement for written or verbal informed consent was waived in view of the retrospective nature of the research.

Consecutive patients who were older than 30 years, had undergone SS-OCT, had controlled diabetes (HbA_1c_ < 8%), did not have high myopia (< -6 diopters) or a history of ocular surgery, intravitreal injections, ocular infection, severe macular edema, and/or epiretinal membrane, and did not have elevated intraocular pressure (>21 mmHg) according to Goldmann applanation tonometry, glaucoma, or proliferative diabetic retinopathy were eligible for inclusion in the study. One eye was selected at random if both eyes in one patient met the study inclusion criteria. In total, 184 eyes were selected for image evaluation, comprising 39 eyes with no diabetic retinopathy, 84 eyes with NPDR, and 61 eyes that had undergone PRP due to severe or very severe NPDR. Eighty-eight eyes of age-matched controls with normal eyes (n = 45) or eyes with mild NTG (n = 43, mean deviation of more than -6 dB on 24–2 mode automated perimetry) were also included. For image quality control, we excluded 66 eyes in which either of 2 blinded examiners was unable to visualize the lamina cribrosa and/or peripapillary tissue clearly. Finally, 141 eyes (1 eye of each patient) were selected (30 eyes of patients with diabetes but without diabetic retinopathy, 66 eyes with NPDR, and 45 eyes in which PRP had been performed) retrospectively from the medical records. As a control, 65 eyes were selected and compared with those in the diabetes group.

Patients with diabetic retinopathy or glaucoma underwent SS-OCT, a visual acuity test, tonometry, and a slit-lamp examination under induced mydriasis by retinal and/or glaucoma specialists. To investigate the effect of time elapsed since PRP on the optic disc and RNFL measurements, the data for the 45 eyes in the PRP group were stratified according to the time interval since PRP. Argon-green (532 nm) laser PRP had been administered about 2000–3000 times, divided into 2–4 sessions per eye, with the distance of at least 1 disc diameter from the major temporal vessel arcade and the optic disc.

### Tomographic analysis by SS-OCT

**Fig 1:**
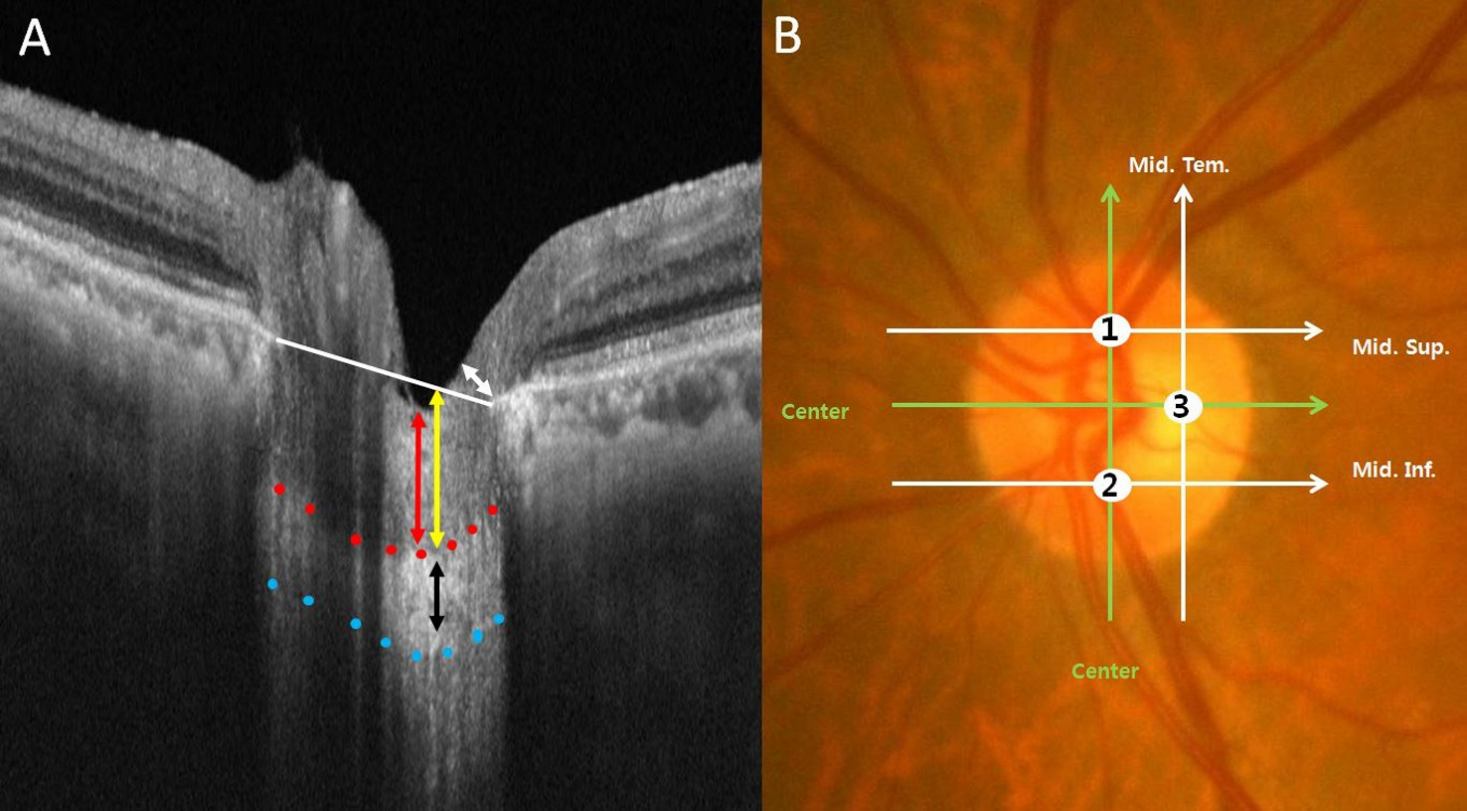
Horizontal cross-sectional B-scan image and photograph of the optic disc. (A) The reference (white) line, representing the shortest distance of the retinal pigment epithelium opening, was set as the baseline for measuring the minimum neuro-retinal rim thickness (white double-arrow) at the optic disc margin, anterior lamina cribrosa depth (yellow double-arrow), prelaminar thickness (red double-arrow), and thickness of the lamina cribrosa (black double-arrow between red and blue dotted line), using swept-source optical coherence tomography. (B) At each point of the mid-superior (1), mid-inferior (2), and mid-temporal (3) areas, the thickness of the lamina cribrosa was measured.

Rather than using conventional OCT scan, we employed SS-OCT (DRI OCT1; Topcon, Tokyo, Japan), which acquires choroid-focused images that allow better visualization of structures lying deeper in the eye, to obtain a three-dimensional (3D) volumetric scan producing OCT images containing consecutive scans with a scan length of 6 × 6 mm in disc image mode and a scan size of 512 × 128. The prelaminar thickness, anterior lamina cribrosa depth (ALD), and thickness of the lamina cribrosa were measured using the caliper tool built into the custom OCT viewer from the mid-superior, mid-inferior, and mid-temporal areas of the line connecting the center of the optic nerve and the Bruch’s membrane opening to determine whether there were any significant differences between the groups (Fig 1). The B-scan image of each measurement point in Fig 1B was selected from among the total 3D data set using the OCT viewer software. The reference line, i.e., the shortest distance of the retinal pigment epithelium opening, was set as the baseline. The measurement points were selected by dividing the total length of the reference line by 2 or 4 depending on the mid-superior, mid-inferior, or mid-temporal point of the disc center (Fig 1B). The anterior laminar cribrosa depth was measured from the reference line to the anterior surface of the lamina cribrosa at each point. At the same point, the prelaminar thickness and thickness of the lamina cribrosa were defined as the minimum vertical length between the anterior and posterior surface of the prelaminar tissue and the anterior and posterior surface of the lamina cribrosa, respectively. The minimum neuro-retinal rim thickness was measured using the thinnest RNFL bundle thickness value at the disc margin of choice, and the average rim thickness of the temporal and nasal sides was used as the superior and inferior rim thickness, whereas the measurements obtained from the temporal side were used as the minimum temporal rim thickness. The peripapillary RNFL thickness was automatically calculated using the software provided from the temporal, superior, inferior, and nasal quadrants of the circle within a radius of 1.7 mm around the center of the optic disc. In addition, using the en face image (EnView version 1.00; Topcon), the horizontal and vertical sizes of the optic disc were measured with the built-in caliper tool, after flattening the retinal pigment epithelium layer, and then averaged for analysis. For cases in which there were no clear boundaries between the tissues around the optic disc and the lamina cribrosa, the consecutive front and back SS-OCT images were analyzed concurrently. Two examiners, blinded to patient information, measured the size of the optic disc and the neuro-retinal rim thickness, prelaminar thickness, ALD, and thickness of the lamina cribrosa using the manual tools provided by the software program. Two measurements from each examiner were averaged to evaluate the final thickness of the lamina cribrosa. In addition, reproducibility was assessed between the 2 examiners.

### Statistical analysis

All data are presented as the mean ± standard deviation. When comparing 2 groups, either the independent *t*-test or the chi-square test was used, depending on the number of samples and on whether the assumption of normal distribution was met. To interpret the primary data collected from 3 or more groups regarding the thickness of a single layer of tissue around the optic disc and of the lamina cribrosa, we performed analysis of variance (ANOVA) and the Kruskal-Wallis test. The Scheffe test or *t-*test was used for post-hoc analysis for further verification.

Univariate and multivariate linear regression analysis was performed to ascertain the relationship between the peripapillary RNFL and the variables obtained using OCT in the patients with diabetes. R-values ≥ 0.1 indicated a weak linear relationship and those ≥ 0.3 indicated a clear linear relationship. Negative values indicated a negative linear relationship and positive values indicated a positive linear relationship. To compensate for the effect of variables such as age, intraocular pressure, and duration of diabetes, the most appropriate multivariate model was selected using a stepwise linear regression method to indicate the relationship between the variables. The intraclass correlation coefficient (ICC) was calculated to verify the reproducibility of each variable manually measured using SS-OCT. The correlation was classified as excellent if the ICC was 0.75 or above, moderate if it was 0.75 to 0.4, and poor if it was 0.4 or below. The statistical analysis was performed using SPSS version 18.0 software (SPSS Inc., Chicago, IL, USA) and p-values < 0.05 were considered to be statistically significant.

## Results

### Patient demographic data

A total of 206 eyes were registered, comprising 33 normal eyes (group I), 30 eyes of patients with diabetes unaffected by diabetic retinopathy (group II), 66 eyes with NPDR (group III), 45 eyes with PRP (group IV), and 32 eyes with NTG (group V). The average age of the entire study cohort was 55.4 ± 10.8 years. Of the 206 eyes included, 112 were in male patients. The average intraocular pressure was 15.7 ± 2.4 mmHg and the average refractive error was -1.13 ± 2.11 diopters. The average mean deviation (MD) of the 129 eyes in which VF24-2 was performed was -1.4 ± 2.0 dB. The average duration of diabetes for groups II, III, and IV was 12.3 ± 7.7 years and the average time elapsed since PRP was 30.5 ± 38.5 months in group IV. The average HbA_1c_ for the patients with diabetes was 7.1 ± 1.2% and there was no difference in HbA_1c_ between groups II, III, and IV (p = 0.309). On SS-OCT, the average diameter of the optic disc was 1511.1 ± 98.9 μm, and the average peripapillary RNFL thickness, neuro-retinal rim thickness, ALD, prelaminar thickness, and thickness of the lamina cribrosa were 198.8 ± 41.6 μm. 96.7 ± 9.5 μm, 215.1 ± 49.7 μm, 465.9 ± 94.0 μm, and 176.0 ± 29.0 μm, respectively.

### Differences in tissue thickness around the optic disc and lamina cribrosa between the PRP and non-PRP groups

There were no statistically significant differences in age, sex, refractive error, intraocular pressure, or size of the optic disc between the groups (Table 1). Although there were no marked differences between groups I, II, and III in terms of average thickness of the peripapillary RNFL or lamina cribrosa as measured using SS-OCT (p = 0.366 and p = 0.858, respectively), there was a significant decrease in the thicknesses of these structures (4.2 μm and 35.8 μm, respectively) when group IV was compared with group III (p = 0.019 and p < 0.001, respectively). There were no significant differences in rim thickness, ALD, or prelaminar thickness between groups III and IV (p = 0.307, p = 0.877, and p = 0.212, respectively). However, there were statistically significant differences in peripapillary RNFL thickness, neuro-retinal rim thickness, ALD, prelaminar thickness, and thickness of the lamina cribrosa between groups IV and V, with differences of 11.2 μm, 42.4 μm, -64.2 μm, 51.5 μm, and 20.2 μm, respectively (p < 0.001, p = 0.001, p = 0.019, p < 0.001, and p = 0.018). Compared with groups III and IV, group V had a thinner peripapillary RNFL, neuro-retinal rim, prelamina, and lamina cribrosa and a larger ALD (Table 1).

**Table 1:** Clinical characteristics of the study cohort.

Characteristic	Total	Group I (normal)	Group II (no DR)	Group III (NPDR)	Group IV (PRP)	Group V (NTG)	p-value^a^
Number of eyes	206	33	30	66	45	32	
Age (years)	55.4 ± 10.8	53.5 ± 8.8	55.7 ± 12.3	56.6 ± 10.4	56.2 ± 9.9	53.8 ± 13.0	0.595^a^
Sex (male/female)	112/94	16/17	18/12	37/29	27/18	14/18	0.566^b^
Refractive error (D)	-1.13 ± 2.11	-0.71 ± 1.82	-0.65 ± 1.94	-1.23 ± 2.34	-1.24 ± 1.71	-1.68 ± 2.48	0.263 ^a^
Intraocular pressure (mmHg)	15.7 ± 2.4	15.6 ± 2.5	16.2 ± 2.5	15.1 ± 2.2	16.0 ± 2.7	16.2 ± 1.9	0.105^a^
DM duration (year)	-	-	6.4 ± 3.6	13.0 ± 7.9	15.0 ± 7.4	-	<0.001^a^
Visual field mean deviation (dB)	-1.4 ± 2.0 (n = 129)	-0.06 ± 1.25 (n = 21)	-0.38 ± 1.20 (n = 10)	-0.56 ± 1.37* (n = 37)	-1.12 ± 1.53* (n = 29)	-3.74 ± 1.45**^,^*** (n = 32)	<0.001^a^
Average disc diameter (μm)	1511.1 ± 98.9	1493.0 ± 103.1	1520.0 ± 98.2	1515.3 ± 97.6	1516.7 ± 103.0	1504.8 ± 95.4	0.787 ^a^
Average peripapillary RNFL thickness (μm)	96.7 ± 9.5	101.3 ± 6.4	101.3 ± 5.9	99.4 ± 8.6 *^,^***	95.2 ± 8.0 *^,**^	84.0 ± 7.2 **^,^***	<0.001^a^
Average neuro-retinal rim thickness (μm)	215.1 ± 49.7	233.5 ± 42.9	231.5 ± 34.4	224.3 ± 41.6 *	210.7 ± 53.5 *	168.3 ± 50.54**^,^***	<0.001^a^
Average lamina cribrosa depth (μm)	465.9 ± 94.0	440.9 ± 61.3	451.7 ± 74.6	455.2 ± 91.7 *	464.6 ± 106.8 *	528.8 ± 101.8 **^,^***	0.001^**a**^
Average prelaminar thickness (μm)	176.0 ± 29.0	177.5 ± 29.3	176.4 ± 24.4	183.2 ± 21.4 *	190.5 ± 23.2 *	139.0 ± 24.0 **^,^***	<0.001^a^
Average LC thickness (μm)	198.8 ± 41.6	212.7 ± 37.4	218.4 ± 44.0	215.0 ± 40.5 *^,^***	179.2 ± 25.5 *^,^**	159.0 ± 14.5 **^,^***	<0.001^a^^`^

DM, diabetes mellitus; DR, diabetic retinopathy; LC, lamina cribrosa; NPDR, non-proliferative diabetic retinopathy; NTG, normal tension glaucoma; PRP, panretinal photocoagulation.

^a^One-way ANOVA.

^b^Chi-squared test.

*p-value < 0.05 compared with NTG group by post-hoc analysis using Tukey’s method.

**p-value < 0.05 compared with NPDR group by post-hoc analysis using Tukey’s method.

***p-value < 0.05 compared with PRP group by post-hoc analysis using Tukey’s method.

**Fig 2:**
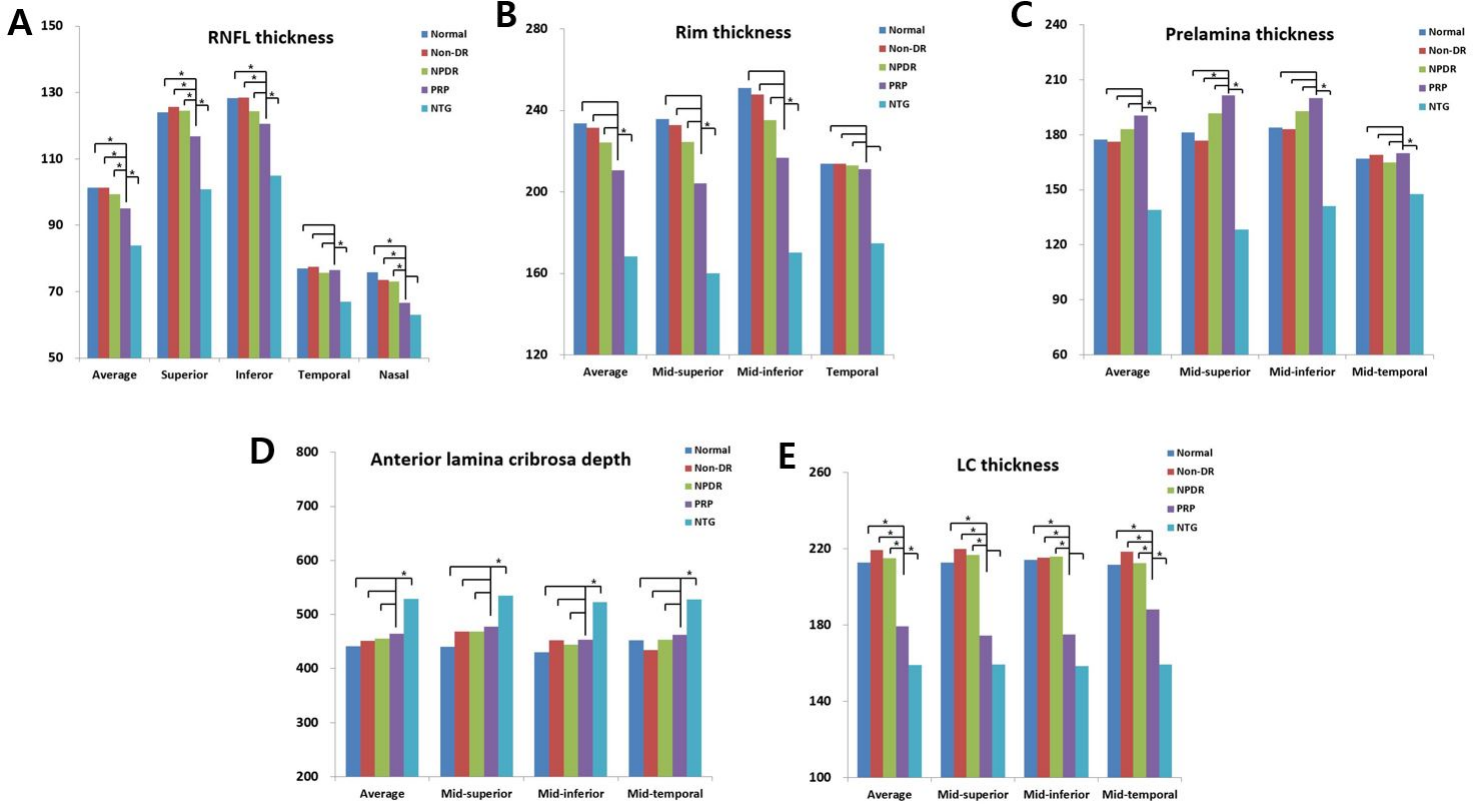
A-E. Comparison of optic disc and lamina cribrosa (LC) thickness in eyes that had undergone panretinal photocoagulation (group VI) with eyes that had not (groups I, II and III) and with eyes that had normal tension glaucoma in 3–4 quadrant areas. *p < 0.05 compared with the PRP group by ANOVA post-hoc analysis using the Scheffe method.

With regard to the morphologic differences in the mid-superior, mid-inferior, and mid-temporal areas (superior, inferior, temporal, and nasal quadrants, respectively, in the case of peripapillary RNFL thickness) of each region measured, the peripapillary RNFL thickness was lower on the superior, inferior, and nasal sides, but not on the temporal side, in group IV when compared with the groups in which PRP was not performed (Fig 2). These features appear to be different from those in patients with NTG, where the thickness values in all quadrants were smaller. However, there were no noticeable differences in rim thickness, prelaminar thickness, or ALD even in the PRP group. The lamina cribrosa was significantly thinner across all regions in group IV than in groups I, II and III; however, the thickness of the lamina cribrosa in group IV was not significantly different from that in group V in the mid-superior and mid-inferior areas after PRP (p = 0.092 and p = 0.068, respectively).

### Factors affecting peripapillary RNFL in patients with diabetic retinopathy

The relationship between peripapillary RNFL thickness and changes in tissues around the optic disc and lamina cribrosa in patients with diabetes was investigated by univariate analysis (Table 2). Peripapillary RNFL thickness had a significant correlation with the presence of diabetic retinopathy and PRP (R = -0.275, p = 0.003). There was also a correlation with time since PRP and thickness of the lamina cribrosa (p < 0.001 and p = 0.013, respectively). However, there was no correlation between peripapillary RNFL thickness and mean age, refractive error, intraocular pressure, duration of diabetes, ALD, or prelaminar thickness. In multivariate analysis, there was a relationship between peripapillary RNFL thickness and both PRP and thickness of the lamina cribrosa (p < 0.001 and p = 0.014, respectively), but not with prelaminar thickness (p = 0.177).

**Table 2:** Univariate and multivariate stepwise linear regression analysis for determining peripapillary retinal nerve fiber layer thickness in patients with diabetes.

Factor	Univariate analysis		Multivariate analysis (corrected R = 0.709)	
	Regression coefficient B	p-value	Standardized coefficient beta	p-value
Group (II, III, and IV)	-0.275	0.003		
Age (years)	-0.067	0.485		
Sex (male/female)	-0.084	0.380		
Refractive error (D)	-0.123	0.195		
Intraocular pressure (mmHg)	-0.029	0.760		
DM duration (years)	-0.057	0.552		
Elapsed time after PRP (months)	-0.611	<0.001	-0.487	<0.001
Visual field mean deviation (dB)	0.145	0.241		
Average disc diameter (μm)	-0.063	0.508		
Average neuro-retinal rim thickness (μm)	0.140	0.140		
Average lamina cribrosa depth (μm)	0.053	0.582		
Average prelaminar thickness (μm)	0.046	0.627	0.157	0.177
Average lamina cribrosa thickness (μm)	0.235	0.013	0.309	0.014

DM, diabetes mellitus; PRP, panretinal photocoagulation.

Regression equation using non-standardized B coefficients: Y = 70.627–0.101X_1(time since PRP)_ + 0.054X_2(average prelaminar thickness)_ + 0.097X_3(average lamina cribrosa thickness)._

**Fig 3:**
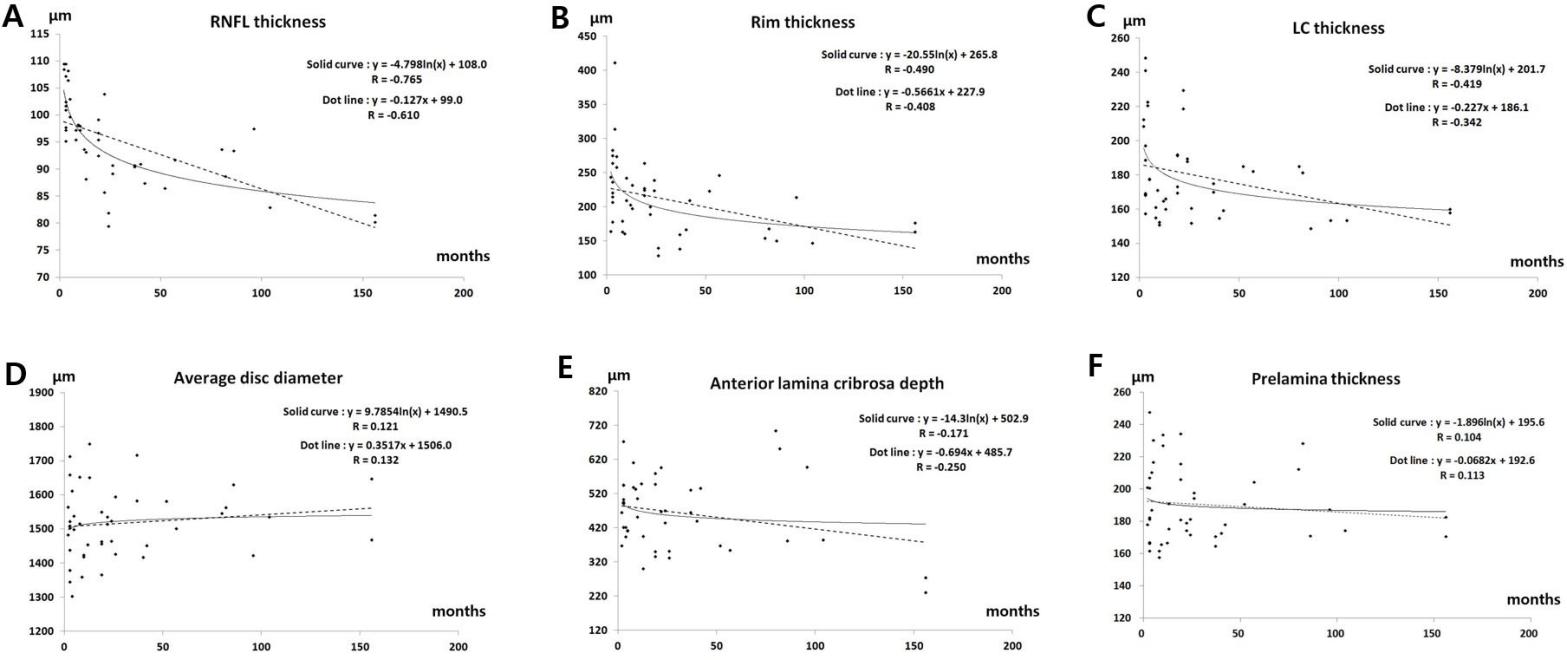
Relationship between neural tissues around the optic disc and time since panretinal photocoagulation. Both logarithmic and linear regression analysis and the R values for linear and logarithmic regression are shown in each graph. (A–C) The retinal nerve fiber layer (RNFL), neuro-retinal rim, and lamina cribrosa (LC) thicknesses showed a good correlation with time since PRP. (D–F) There were no correlations between time since PRP and average disc diameter, anterior LC depth, or preretinal thickness.

Table 3 shows the results for the 3 subgroups created on the basis of time since PRP. We found that the peripapillary RNFL and neuro-retinal rim were significantly thinner according to the amount of time elapsed since PRP (p < 0.001 and p = 0.006, respectively). Fig 3 shows that there was a significant negative correlation between time since PRP and thicknesses of the RNFL, neuro-retinal rim, and lamina cribrosa (R = -0.765, -0.490 and -0.419, respectively, in logarithmic regression). The relationship between these 3 variables appears to be more logarithmic than linear. Further, there was a relatively weak linear correlation between time since PRP and the size of the optic nerve, ALD, and prelaminar thickness (R = 0.132, -0.250 and 0.113, respectively, in linear regression).

**Table 3:** Clinical characteristics in subgroups of patients with panretinal photocoagulation (group IV) according to time since procedure.

Characteristic	PRP (<1 year)	PRP (1–3 years)	PRP (≥3 years)	p-value
Number of eyes	19	13	13	
Age (years)	54.5 ± 13.2	57.4 ± 7.0	57.5 ± 6.7	0.630^a^
Sex (male/female)	12/7	7/6	8/5	0.870^b^
Refractive error (D)	-1.47 ± 1.77	-1.87 ± 2.03	-0.29 ± 0.62	0.067 ^a^
Intraocular pressure (mmHg)	15.8 ± 3.0	15.9 ± 2.4	16.5 ± 2.6	0.804^a^
DM duration (years)	12.1 ± 6.6	12.5 ± 5.4	21.9 ± 6.1	<0.001^a^
Time since PRP (months)	4.8 ± 2.7	19.9 ± 4.8	78.9 ± 41.3	<0.001^a^
Visual field mean deviation (dB)	-1.43 ± 1.2 (n = 12)	-1.33 ± 1.76 (n = 9)	-0.41 ± 1.68 (n = 8)	0.315^a^
Average disc diameter (μm)	1496.8 ± 112.4	1519.2 ± 101.7	1543.5 ± 90.5	0.461^a^
Average peripapillary RNFL thickness (μm)	101.8 ± 5.0	91.6 ± 6.8	88.9 ± 5.1	<0.001^a^
Average neuro-retinal rim thickness (μm)	236.4 ± 61.6	206.1 ± 37.8	177.8 ± 33.7	0.006^a^
Average lamina cribrosa depth (μm)	488.3 ± 77.0	439.3 ± 103.7	455.1 ± 143.1	0.422^a^
Average prelaminar thickness (μm)	194.2 ± 28.1	190.2 ± 19.5	185.5 ± 19.1	0.597^a^
Average LC thickness (μm)	186.7± 30.6	181.0 ± 23.3	166.5 ± 13.5	0.084^a^

DM, diabetes mellitus; PRP, panretinal photocoagulation; RNFL, retinal nerve fiber layer.

^a^Kruskal–Wallis test.

^b^Chi-squared test.

### Reproducibility of tomographic variables using SS-OCT

The ICC was calculated for each variable in all 5 groups and indicated good inter-rater agreement for the size of the optic nerve, the rim thickness, ALD, and the thickness of the prelamina and lamina cribrosa (Table 4).

**Table 4:** Interobserver reproducibility of average disc diameter, retinal nerve fiber layer thickness, rim thickness, lamina cribrosa depth, prelamina tissue thickness, and average lamina cribrosa thickness measurements in all eyes.

Characteristic	Mean value	Observer 1 (mean ± SD)	Observer 2 (mean ± SD)	ICC	95% CI
Average disc diameter	1511.1 ± 98.9	1509.0 ± 131.0	1515.0 ± 115.7	0.863	0.820–0.896
Average neuro-retinal rim thickness	215.1 ± 49.7	220.1 ± 57.3	210.1 ± 49.9	0.972	0.963–0.979
Average lamina cribrosa depth	465.9 ± 94.0	472.2 ± 94.1	459.6 ± 96.5	0.833	0.781–0.873
Average prelaminar thickness	176.0 ± 29.0	174.9 ± 31.0	177.1 ± 29.0	0.927	0.904–0.945
Average lamina cribrosa thickness	198.6 ± 41.1	201.7 ± 36.9	195.5 ± 48.8	0.891	0.856–0.917

CI, confidence interval; ICC, intraclass correlation coefficient; SD, standard deviation.

## Discussion

The change in the shape of the optic disc in patients with diabetes has been investigated in previous studies, but the results reported have been inconsistent [3,4,11,12]. In a longitudinal study of 100 patients in whom stereoscopic disc photography was used, Johns et al. showed no significant change in the shape of the optic disc after PRP in patients with diabetic retinopathy [11]. In contrast, in a recent cross-sectional study, Lim et al. reported that changes in the optic disc indicating optic neuropathy were different from those in patients with glaucoma and that those changes became prominent after PRP [3,12]. However, methods for objective and quantitative analyses may have not been available to the investigators in previous studies, most of which relied on slit-lamp and/or fundus photography for funduscopic examination, and the results obtained using these methods are largely dependent on the subjectivity of the examiner [4,12].

The advent of advanced technology, such as OCT, has led to an increasing number of quantitative studies using tomographic variables such as peripapillary RNFL thickness, disc size, and thickness of the lamina cribrosa. This technology has allowed more objective visualization of the damage to neural tissues in patients with diabetes and glaucoma [12–23]. Dijk et al. argued that diabetes is not only associated with changes in the vasculature but also with neuro-degeneration, given that the functional reduction in patients with early-stage diabetic retinopathy is accompanied by significant thinning of the ganglion cell layer and the RNFL [24]. Further, previous studies have shown that the decrease in peripapillary RNFL thickness becomes more marked with progression of diabetes [25]. It seems that PRP in patients with diabetes decreases the thickness of these neural tissues further, leading to a reduction in RNFL thickness around the optic disc [26,27]. Measurement of RNFL thickness directly is difficult in the presence of retinal edema, which is common in diabetes; thus, if tissues around the optic disc and lamina cribrosa are measured at the same time, it would be possible to obtain a better understanding of the changes that occur in the neural tissue and optic disc in diabetes [26]. However, with the exception of peripapillary and retinal RNFL thickness, the changes in the tomographic variables described here have only been reported previously in the context of development, type, and progression of glaucoma.

A potential mechanism underlying the glaucomatous changes in tissues around the lamina cribrosa is as follows. High intraocular pressure reduces the thickness of the prelaminar tissue by pushing on the lamina cribrosa and increasing its depth, which causes changes in both structures [22,28]. The thickness of the lamina cribrosa changes in response to blocking of the flow of axoplasm as well as ischemia, which in turn leads to mechanical damage as well as deformation because of the increased intraocular pressure [22,28,29]. Thus, the tissues around the RNFL and lamina cribrosa may be damaged by ischemia, neuro-degeneration, and PRP in patients with diabetes. However, SS-OCT was only introduced recently, so 3D images providing detailed information about the tissue around the optic nerve are still lacking, and standard thickness values have to be defined for each tissue in both the general population and the diabetic patient population.

Several studies of ALD, prelaminar thickness, and lamina cribrosa thickness in patients with glaucoma have been reported. Although it is known that intraocular pressure increases and glaucomatous changes occur in the optic disc and tissues around the lamina cribrosa, the sequence of events and the underlying mechanism are still debated [30–33]. In this study, loss of the RNFL in the retinal area may have influenced the peripapillary RNFL and optic disc measurements, including the thicknesses of the neuro-retinal rim and lamina cribrosa in the patients with PRP. However, the ALD and prelaminar thickness did not change to a great extent after PRP. The mechanism underlying this phenomenon in patients with diabetes is thought to lie in proliferation of glial cells around the damaged nerve fiber, resulting in reorganization of the damaged regions [34]. In the present study, prelaminar thickness appeared to be increased slightly, albeit not significantly so, despite a decrease in the amount of neural tissue present after PRP.

The studies performed to date have included a variety of patient populations, study methods, and measuring equipment; thus, normal values for the variables included in our present study have yet to be agreed on. The ALD is one of the few variables for which average normative data have been suggested, i.e., 341.8 μm for patients with pseudoexfoliation syndrome and 306.3 μm for the opposite normal eye, while the value for patients with glaucoma has been reported to be 469.2 μm in another study [16,23]. In this study, the ALD for the normal eyes was about 440.9 μm and that for the group with prior PRP treatment was about 464.6 μm, with no significant difference between the 2 groups; the ALD was 528 μm in the group with NTG. The average thickness of the lamina cribrosa has been shown to be 219.5–254 μm for normal eyes and 197.8–147.2 μm for eyes with NTG [18,19,23,35]. In normal eyes and eyes of patients with mild NTG, the average thicknesses of the lamina cribrosa at the mid-superior, mid-inferior, and mid-temporal areas were 212.7 ± 37.4 μm, and 159.0 ± 14.5 μm, respectively; there was no statistically significant difference in any area (p < 0.410), which is similar to the previous reports.

Thinning of the RNFL in patients with prior PRP treatment was significant in the superior, inferior, and nasal quadrants where the laser beam was directly applied. Thinning of the RNFL and rim thickness without a decrease in prelaminar thickness or an increase in ALD is thought to affect the general shape of the optic nerve, which in turn may lead to changes in the thickness of the lamina cribrosa as compared with that in patients with NTG. The possible mechanisms include the following. First, the ischemic change in the tissue around the lamina cribrosa and the optic disc caused by diabetes and the laser, as well as the loss of extracellular matrix, may have caused the reduction in tissue thickness. Second, the thin fiber axons passing through the lamina cribrosa may cause backward movement and thinning of the lamina cribrosa, but there was no clear increase in ALD in the present study. Third, the reorganization of prelaminar thickness and reduction in RNFL thickness may cause an increase in sensitivity to pressure and blood flow in the tissues around the lamina cribrosa, thus causing the change in lamina cribrosa thickness. Fourth, changes such as a reduction in thickness of or blood flow to the choroid after PRP [36] are thought to change the characteristics of the lamina cribrosa. Finally, there is a possibility that the retinal blood vessels that pass through the thin lamina cribrosa would affect the extent of disease progression in patients with diabetic retinopathy and the structures around the lamina cribrosa in patients who undergo PRP.

We also found that the thickness of the tissues around the optic disc and lamina cribrosa was decreased depending on the duration of diabetes and time since PRP, and that even after all variables were adjusted for, the time since PRP and lamina cribrosa thickness still seemed to have an influence on reduction of the peripapillary RNFL after PRP. In addition, when a scatter diagram was drawn for the OCT variables according to time since PRP, the reduction in the logarithmic shape appeared to be more than that in the linear shape. Thus, it can be presumed that after PRP, the thicknesses of the RNFL, the neuro-retinal rim, and the lamina cribrosa decrease dramatically in the early days and then stabilize with the passage of time. It appears that PRP damages the RNFL and nearby capillary veins, which in turn leads to a change in and reorganization of the elastin and collagen that constitute the lamina cribrosa, resulting in a rapid decrease in the thickness of the lamina cribrosa early after PRP before slowing down at a later stage [34,37,38].

One of the interesting observations in this study was that the group of patients with NTG showed a uniformly thinner peripapillary RNFL, neuro-retinal rim, and lamina cribrosa in all regions when compared to the patients without NTG; in contrast, the group of patients with PRP had thinner values for these parameters in the superior, inferior, and nasal quadrants, but not in the temporal quadrants, when compared to those without PRP. It is thought that the asymmetric reduction in thickness between the RNFL and the lamina cribrosa may be caused by the radial running of the RNFL, which is mainly distributed throughout the macula, passing by the section where the laser is applied. In contrast, the lamina cribrosa located on the temporal side remains relatively undamaged by the laser. This observation supports the theory of an acquired change in the thickness of the lamina cribrosa, and that a change in the thickness of the RNFL or peripapillary tissue would directly relate to a change in the lamina cribrosa [34,35,39–41].

This study has some limitations. First, it is a retrospective cross-sectional analysis, and we did not directly observe changes in the tissues around the optic disc or lamina cribrosa during progression of diabetes after PRP. Second, we excluded patients with proliferative diabetic retinopathy to minimize confounding variables, such as damage to the nerve caused by complications related to microvascular disease, damage to the optic nerve caused by neuro-degeneration, poor diabetes control, severe retinal edema, and retinal bleeding. While we compared the direct effects of PRP on various tissues, we did not observe the effects of PRP on the optic disc as the disease progressed to proliferative retinopathy. Third, there may have been some selection bias due to exclusion of a large proportion of eyes (24.3%) because of poor peripapillary images, even with choroid-focused SS-OCT. Finally, the changes in the tissue around the optic disc and lamina cribrosa, depending on variables such as intraocular pressure, refractive error, and age, were not found to be statistically significant, although they have been found to be significant in previous studies. This discrepancy might be related to the effect of the retina and choroid blood flow on glaucoma and the changes in capillary perfusion in diabetic retinopathy, which were not included in our study [42–45]. The variables that should be adjusted for, e.g., patient age and sex, were not obvious at this point; hence, an adjusted p-value was not considered in this exploratory study. Therefore, prospective longitudinal studies with large samples and OCT angiography data are needed to determine whether our present findings reflect the presence of diabetes, the ocular complications of diabetes, the treatment provided for diabetes, or simply the limited number of patients.

In conclusion, we found in this study that PRP appeared to be associated with significant thinning of the peripapillary RNFL, neuro-retinal rim, and lamina cribrosa but had no significant effects on prelaminar tissue thickness or ALD. PRP was associated with characteristic peripapillary morphologic changes, and our results suggest that these changes are essentially different from typical glaucomatous changes in that they are characterized by a pattern of decreased thickness of the RNFL, prelamina, and lamina cribrosa, as well as an increased ALD.
